# Data-Driven Dispatching Rules Mining and Real-Time Decision-Making Methodology in Intelligent Manufacturing Shop Floor with Uncertainty

**DOI:** 10.3390/s21144836

**Published:** 2021-07-15

**Authors:** Liping Zhang, Yifan Hu, Qiuhua Tang, Jie Li, Zhixiong Li

**Affiliations:** 1 Key Laboratory of Metallurgical Equipment and Control Technology of Ministry of Education, Wuhan University of Science and Technology, Wuhan 430081, China; zhangliping@wust.edu.cn (L.Z.); huyifan@wust.edu.cn (Y.H.); 2 Hubei Key Laboratory of Mechanical Transmission and Manufacturing Engineering, Wuhan University of Science and Technology, Wuhan 430081, China; 3 Centre for Process Integration, Department of Chemical Engineering and Analytical Science, The University of Manchester, Manchester M13 9PL, UK; jie.li-2@manchester.ac.uk; 4 Yonsei Frontier Lab, Yonsei University, 50 Yonsei-ro, Seodaemun-gu, Seoul 03722, Korea; zhixiong.li@yonsei.ac.kr; 5 Faculty of Mechanical Engineering, Opole University of Technology, 76 Proszkowska St., 45-758 Opole, Poland

**Keywords:** data-driven, machine learning, dispatching rules, offline training, online decision-making

## Abstract

In modern manufacturing industry, the methods supporting real-time decision-making are the urgent requirement to response the uncertainty and complexity in intelligent production process. In this paper, a novel closed-loop scheduling framework is proposed to achieve real-time decision making by calling the appropriate data-driven dispatching rules at each rescheduling point. This framework contains four parts: offline training, online decision-making, data base and rules base. In the offline training part, the potential and appropriate dispatching rules with managers’ expectations are explored successfully by an improved gene expression program (IGEP) from the historical production data, not just the available or predictable information of the shop floor. In the online decision-making part, the intelligent shop floor will implement the scheduling scheme which is scheduled by the appropriate dispatching rules from rules base and store the production data into the data base. This approach is evaluated in a scenario of the intelligent job shop with random jobs arrival. Numerical experiments demonstrate that the proposed method outperformed the existing well-known single and combination dispatching rules or the discovered dispatching rules via metaheuristic algorithm in term of makespan, total flow time and tardiness.

## 1. Introduction

Intelligent manufacturing shop floors usually use the cutting-edge technologies like IoT, cloud manufacturing, agent-based techniques, and big data to convert typical production resources such as workers, machines, materials and orders into smart manufacturing objects [1]. Smart manufacturing objects create an intelligent manufacturing shop floor with huge production data. Real-time production data, which can show the scheduling efficiency and so on, plays more and more important roles in today’s competitive manufacturing industry. There are quite a number of available approaches to collect production data available. But many enterprises are still troubled because there are no effective data process methods. In fact, the production parameters like processing time, the distribution of job arrival, hint some kind of regularity in an intelligent. On this account, once this regularity of the production data is discovered, the scheduling knowledge can be easy to apply into the real-time decision making, which serves as the primary motivation of this paper.

Meanwhile, lots of disturbance in the intelligent shop floor will cause the production fluctuation or interruption. In order to keep the stability of production process, according to the state of the art, there are three categories of optimization methods [2,3,4,5,6,7,8,9,10]: conventional deterministic methods, meta-heuristic algorithms and dispatching rules. Conventional deterministic methods, including branch and bound, gradient free methods, can guarantee global convergence. Meta-heuristic methods, including genetic algorithm, cross entropy method, teaching-learning based optimization algorithm have been proven effective and efficient in searching high-quality solutions during reasonable time. However, computational time of conventional deterministic methods increases exponentially and the lower bound may not be obtained during polynomial time as the size of the problem increases. meta-heuristic algorithms have not been applied directly to real manufacturing for the lack of convenience and instantaneity [3]. therefore, these methods have been studied successfully, but far from the practical application for the instantaneity and low computability in the intelligent shop floor.

Generally speaking, dispatching rules have been widely used in real intelligent scheduling because they have many advantages such as low computational threshold and convenient implementation and can provide a pretty good solution timely [11,12]. According to no free lunch theory, many different dispatching rules have been used in scheduling job shops, but no single dispatching rule dominates the others for a given performance criterion under all conditions [13]. Currently, the dispatching rules usually play a good performance in a steady state behavior of the system. With changes of the production scenario over time, the proper dispatching rules cannot be self-adaptatively to match the current scenario.

Thus, we consider a novel closed-loop scheduling framework to solve intelligent scheduling with uncertainty. Firstly, the scheduling knowledge hidden in the historical production data are extracted using improved gene express program (IGEP). IGEP is proposed to discover appropriate dispatching rules which can match the current intelligent shop floor scenarios. Then, real-time decision-making method calls newly appropriate dispatching rules for assigning jobs to machines timely and stores the production data into database.

The contribution of this paper is as follows:

(1) This paper proposes an improved IGEP approach to extract the appropriate scheduling knowledge.
(2) An efficient real-time decision-making approach is proposed to respond the disturbance timely.
(3) The appropriate dispatching rules is discovered from the historical production data.

The remainder of this paper is organized as follows. Section 2 gives a brief review of dispatching rules mining and online decision-making method. Section 3 gives the motivations of this paper. The problem statement is described in Section 4. Section 5 provides the framework for data-driven dispatching rule mining and online decision-making. The detail of offline training method is drawn in Section 6. The results of numerical experiments are given in Section 7. Finally, conclusions follow in Section 8.

## 2. Literature Review

The typical production scheduling problems have been studied about 60 years in both academic and industrial environments [14]. Nowadays, the scheduling should deal with a smart manufacturing system supported by novel and emerging manufacturing technologies such as mass customization, Cyber-Physics Systems (CPS) [15], Big Data, the Internet of Things (IoTs), Artificial intelligence (AI), Digital Twin [16], and SMAC (Social, Mobile, Analytics, Cloud) [17]. The scheduling research needs to shift its focus to intelligent scheduling modeling and optimization. The main problem of intelligent scheduling is that lots of disruption events will cause the production fluctuation or interruption. Currently, there are three categories of dynamic scheduling [3,4,5]: active scheduling, robust scheduling and pro-active scheduling, to respond the disturbances. Predictive-reactive scheduling is the most common dynamic scheduling approach used in manufacturing systems [2,4]. It is a scheduling/rescheduling process in which schedules are revised in response to real-time events. Due to the real-time requirement, dispatching rules with lower computational threshold and easy implementation have been widely employed in the intelligent shop floor [6,14]. We summaries the related references and divide into three aspects: choosing simple or combinational dispatching rule directly, selecting the superior dispatching rule via meta-heuristic Algorithm, and mining the efficient dispatching rules.

A dispatching rule is used to select the next job shop to be processed from the set of jobs waiting at a shop floor. Panwalkar and Iskander [18] summarized over 100 priority dispatching rules as early as 1977 for production scheduling. Ho and Tay [19] employed suitable parameters and operator spaces for evolving efficient dispatching rules using genetic programming for solving flexible job shop scheduling problems. Rajendran and Holthaus [20,21] designed several efficient composite dispatching rules for job shop problem. Marko and Domagoj [21] collected a large number of dispatching rules and tested them on nine scheduling criteria and four problem types with various machine and job heterogeneities. The results showed that different dispatching rules were suited for solving different scheduling criteria. It has been observed that no single dispatching rule performs well for all of flow time, tardiness of jobs and other regular and non-regular performance measures particularly in the dynamic environment of job shop scheduling [22,23,24,25,26].

In order to improve the performance of the dispatching rules, Bergmann et al. [27] briefly reviewed the basics of the compared classification methods, including K-nearest neighbors algorithm, Naïve Bayes classifier, support vector machines [28], classification and regression tree, artificial neural networks, in job scheduling problem. Domagoj and Kristina [29] investigated the use of genetic programming in automated synthesis of scheduling heuristics for an arbitrary performance measure. Nguyen et al. [30] proposed genetic programming to discover new dispatching rules automatically for the single objective job shop scheduling problem. Liping Zhang et al. [3,31] proposed the improved GEP to generate the dispatching rules automatically for the energy consumption criteria. Sungbum and Seokcheon [32] addressed the dynamic single-machine scheduling problem for minimization of total weighted tardiness by learning of dispatching rules from schedules. These appropriate discovered dispatching rules can play a good performance in the specific environment. However, the intelligent shop floor will face with a complicated environment, such as job insertion, random jobs arrival. The scheduling scenarios always change over time.

Meanwhile, many dispatching rules perform well on specific scenarios [22]. Some researchers start to focus on selecting the superior dispatching rule via Hyper-Heuristic Algorithm to obtain good performance. Mouelhi-Chibani and Pierreval [33] proposed neural networks approach to select the most suited dispatching rule. The selection is made in accordance with the current system state and the workshop operating condition parameters. Korytkowski et al. [26] developed evolutionary simulation-based heuristics to construct near-optimal solutions for dispatching rule allocation, which to decide the appropriate rules to different environment. Heger et al. [34] investigated the use of Gaussian process to switch appropriate dispatching rules in a stochastic, dynamic job-shop scenarios. Zhang and Roy [35] proposed a semantics-based dispatching rule selection system for job shop scheduling to generate a combination of dispatching rules given randomly selected combination of production objectives. Luo [36] proposed six composite dispatching rules and developed a deep Q-network to promote the rules with higher Q-values at each rescheduling point. The results confirmed that these methods were superior to the single or composite dispatching rules. One drawback for selecting dispatching rules is that the performance of these methods mainly depends on the pre-set dispatching rules. Therefore, this paper mainly focuses on extracting the appropriate dispatching rules to response the real-time event for the predictive-reactive scheduling problem.

## 3. Motivations

Nowadays, intelligent devices have been widely appeared in manufacturing enterprises. At the same time, the data acquisition hardware and information software are also extensively applied for collecting and storing production data in the intelligent shop floor. Therefore, big data are generated and stored into memory space as different data structure. As we known, these data maybe hint the potential regularity of production parameters.

In order to understand and grasp the current potential relationship among production parameters in manufacturing process, we investigate some intelligent shop floors, such as a machining workshop for processing stereo-garage. It is worth to notice that most of managers could realize and try to take full use of the potential value of these data. They try to analyze these production data and report the statistic results like machine utilization, etc. Generally, they only collect and employ the data for reporting the actual production schedule, the quantity of finished jobs and some statistical statement about the actual situation. Otherwise, in order to realize the high production efficiency, the managers expect the intelligent shop floor to keep working all the day without interruption. In fact, random jobs arrival and machine breakdown may cause a huge influence on the original production plan or the whole production line. Therefore, the managers with their rich production experience and management often modify the original plan, even shut down the whole production line to adapt to the uncertain shop floor environment.

**Figure 1:**
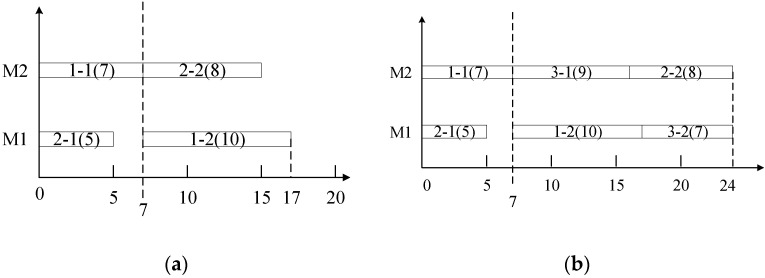

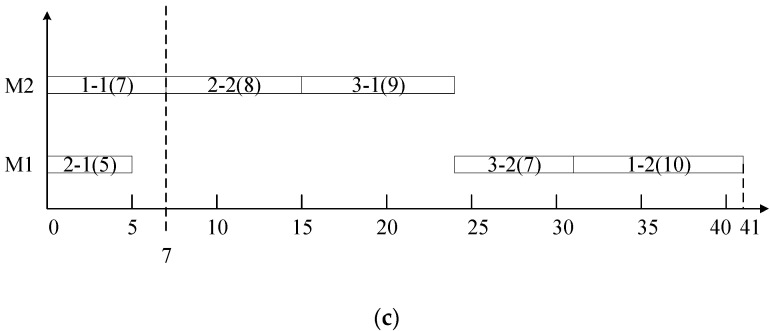
A simple example. (**a**) The Gantt Chart via SPT at time 0. (**b**) The Gantt Chart via LPT at time 7. (**c**) The Gantt Chart via SPT at time 7.

These production experience or management can be usually regard as the scheduling knowledge, such as shortest processing time (SPT) rules, which schedules the operation with shortest processing time first. In most situations, the managers can make a good production plan, but not the best one by their rich production experience or management. In fact, these scheduling knowledge are helpful to improve the scheduling efficiency. Take a simple dynamic job shop scheduling problem as an example, there are 3 jobs and 2 machines with 6 operations. Job 1 and Job 2 arrive the shop floor at time 0. Job 3 arrives the shop floor at time 7. The processing time of Job 1, Job 2 and Job 3 is {7, 10}, {5, 8}, {9, 7}. At time 0, the Gantt Chart is generated via shortest processing time (SPT) rule in Figure 1a. At time 7, the Gantt Chart is generated via longest processing time (LPT) in Figure 1b and SPT in Figure 1c. The performance measure is makespan.

From Figure 1c, the scheduling performance rapidly deteriorates at the first rescheduling point when the dispatching rule remains SPT. In contrast, an appropriate dispatching rule in Figure 1b has a good scheduling performance at this rescheduling point. Actually, managers often modify the original scheduling plan according to their scheduling knowledge. These scheduling knowledges play a critical factor to guarantee and improve the scheduling performance. Therefore, it is very important to explore more efficient dispatching rules with the potential regularities of the key production parameters.

## 4. Intelligent Job Shop Scheduling Problem Statement

In the intelligent manufacturing system, the intelligent shop floor enables to sense dynamic manufacturing environment and react to disturbances agilely. For sensing dynamic manufacturing environment, most research focus on estimating the future trends of jobs arrival or machine failures via regression analysis [37,38,39,40]. This process can ensure the scheduling scheme have the fault tolerant capability. The scheduling scheme may reserve time redundancy based on the predict results and the redundancy can arrange the disturbance events perfectly. For reacting to disturbances agilely, the dispatching rules have been proven to be effective. Most of these dispatching rules come from experience or mining via genetic program or gene expression program.

Generally, the scheduling scheme is generated in two serial phases: the prediction phase and the reaction phase. In the intelligent shop floor, the future trends of dynamic events are commonly predictable according to the historical data. After the prediction phase, the reaction phase employs redundancy or optimization methods to generate the scheduling scheme to fit the current shop floor environment. This method really can enhance the agile responsiveness and make full use of the historical data to improve the production stability. However, the independence of these phases may incur greater intermediate deviation. In fact, extracting dispatching rules from the historical data may integrate these phases efficiently together and reduce the intermediate deviation. Main idea of this paper is to mine the dispatching rule from the related historical data, rather than the benchmark data or the experience combination.

The jobs arrive at the intelligent manufacturing system dynamically over time [37]. The distribution of job arrivals is often various in the different type manufacturing environments. Normally, the distribution of job arrivals process closely follows a Poisson distribution. Hence, the time between job arrivals closely follows an Exponential distribution [41,42,43,44]. As the jobs arrive, the original scheduling scheme more and more deviates from the actual environment. When the scheduling deviation is unacceptable, the rescheduling strategy is applied to generate the new scheduling scheme.

Rescheduling needs to address two issues: how and when to react to the unacceptable deviation [2]. Regarding the first issue, complete rescheduling regenerates a new schedule from scratch and might, in principle, be better in maintaining near-optimal solutions. But these solutions require prohibitive computation time. Therefore, in order to keep the near-optimal solutions and obtain the acceptable computation time, this paper combines the complete rescheduling strategy and the dispatching rules via extracting from related historical data. Regarding the second issue, a periodic rescheduling policy is presented for continuous processing in a dynamic environment.

At each rescheduling point, the problems can be simplified mathematically as follows. There are a set of jobs J={1,…,n}, a set of operations $O=\left\{1,\ldots ,o_{j}\right\}$, and a set of machines M={1,…,m}. The set of jobs contains four types of jobs, finished jobs J_1={1,…,n_1}, being processed jobs  J_2={1,…,n_2}, unprocessed jobs J_3={1,…,n_3} and new jobs J_4={1,…,n_4}. Note that, the set of rescheduling jobs should contain the jobs with unprocessed operations  J_2={1,…,n_2}, the unprocessed jobs  J_3={1,…,n_3} and the new jobs  J_4={1,…,n_4}. All the operations of the unprocessed jobs and the new jobs should be rearranged at each rescheduling point. But just the unprocessed operation of the unprocessed jobs should be rearranged at each rescheduling point. The available time of each machine must be not less than the rescheduling point and the completed time of the being processed jobs. The rescheduling operations must be processed on machine when the machine is available. Each machine can process at most one operation at a time [45]. The problem is to find a feasible unprocessed operations sequence that satisfies the above constraints and minimizes the scheduling efficiency.

For the intelligent job shop problem, the schedule efficiency is considered in evaluating the solutions and is defined as the total time required in processing all of the jobs. During the planning period, the schedule efficiency is measured by the makespan, flow time and tardiness. Makespan traditionally is defined as the total time that is required to process a group of jobs. To fit the intelligent scheduling environment, this definition is modified so that the group of jobs includes all jobs scheduled at a scheduling point. Tardiness is defined using the traditional approach, namely, the difference between the completion time and due date for each job in which the completion time occurs after the due date.

Objective function is as follow.

Makespan:

$$\min mak=\underset{all\;i}{\max}C_{i}$$

Flow time:

$$\min FT=\overset{n}{\underset{i=1}{\sum }}\left(C_{i}-AT_{i}\right)$$

Tardiness:

$$T=\overset{n}{\underset{i=1}{\sum }}max\left(DD_{i}-C_{i},0\right)$$

where, *n* is the total number of jobs. *AT_i_* is the arrival time of job *i*. *DD_i_* is due date of job *i*. *C_i_* is the complete time of job *i*.

## 5. Data-Driven Dispatching Rule Mining and Decision-Making Framework

Along with the rise of IoT technologies, cloud computing, big data analytics, AI, and other technological advances, came the age of big data [46]. In manufacturing, effective analysis of big data enables manufacturers to deepen their understanding of customers, competitors, products, equipment, processes, services, employees, suppliers and regulators. Big data can help manufacturers to make more rational, responsive, and informed decisions, and enhance their competitiveness in the global market [47]. Moreover, intelligent shop floor should enable to sense dynamic manufacturing environment and agilely react to disturbances [48].

As mentioned above, dispatching rules are often suggested to schedule production timely. Though numerous dispatching rules exist, unfortunately no dispatching rule is known to be superior to others [49]. Therefore, we formulate IGEP which explores the newly dispatching rules from the historical production data. Main advantage of this approach enables to mine the potential relationship among the manager’s expectation and production parameters from the historical data, such as the relationship between makespan and the processing time. It is helpful to explore the potential regulations from the historical data and explicit potential regulations by mathematical expression.

**Figure 2:**
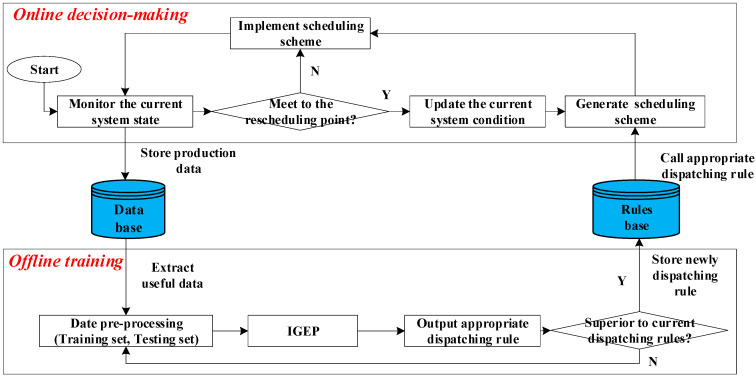
The framework of the proposed approach.

Figure 2 illustrates the framework of the proposed approach. The framework is centered on a novel dispatching rules mining method to improve global performance by using IGEP. The framework takes advantages of the potential of historical data and the convenience and instantaneity of dispatching rules. At each rescheduling point, the appropriated dispatching rule enables to generate a near-optimal scheduling scheme for real time response of the disruption events. At the same time, IGEP is designed to discover the newly appropriated dispatching rule from the historical production data.

During the planning period, production data will be stored into database. The appropriated dispatching rule is updated by IGEP with the state of shop floor. When the rescheduling point occurs, the current system updates the workshop operating condition parameters immediately and calls appropriate dispatching rules to generate the new proper scheduling scheme timely. Then, the original scheduling scheme is deleted and the intelligent shop floor will execute the new scheduling scheme.

## 6. Offline Training Method

The literatures have proved that the suitable dispatching rules at different scenario have better performance than the consistent one dispatching rule. Since different rules are suitable for different scenarios, it is hard for the decision makers to select the best rule at a specific time point [36]. It is almost impossible to design a general appropriate dispatching rule for all the production scenario. As we known, the production environment of the intelligent shop floor, such the layout of shop floor, the machines status, the jobs type, is relatively internally consistency and regularity during an interval of time. Thus, this paper aims to mine the potential dispatching rules from the historical production data during a certain period, which may owe the optimal performance for the current scenario. If the potential dispatching rules are explored successfully, it’s exactly applied into the current rescheduling point and it can be self-study over time.

In the big data age, empowered by the new ITs, manufacturer’s ability to collect, store and process data is significantly enhanced [33]. This paper assumes that the historical production data are stored into the date base in near real-time. These data are randomly divided into two set: training set and testing set. The training set, about 80 percent of the total data, is used to discover the new efficient dispatching rules via IGEP. The testing set, about 20 percent of the total data, is applied to verify the performance of IGEP. If the new discovered dispatching rules are superior the original rules in the rules base, the new discovered dispatching rules will cover the original rules.

One of the greatest strengths of this method is that this process does not consume the online production time when it constructs the rules base. Moreover, IGEP owns the merits of artificial intelligence via self-study and unsupervised learning, and also the advantages of swarm intelligence via population diversity and convergence.

### 6.1. Data Pre-Processing

During the evolution of IGEP, the performance of the evaluation function with the evolved direction has a strong relation to the training data [50]. The proper training data is helpful to enhance the high prediction accuracy. The parameters related to jobs and machines will be stored into the database completely at each rescheduling point. These data are defined as one training instance.

Note that in the training stage, in order to enhance exploration, the offline training method at beginning rescheduling point isn’t activated until the pre-set rescheduling point is meet. On the other hand, the intelligent shop floor will produce huge amounts of data over time. It is impossible and worthless to train the IGEP from all the production data. This paper chooses the production data among the latest several rescheduling points as the training data. This can grantee the discovered dispatching rules to adapt to the current specific time point.

### 6.2. The Improved Gene Expression Programming

Gene expression programming [50,51] is an evolutionary artificial intelligence technique developed by Ferreira (2001), and has been used with increasing frequency to address symbolic regression, time series prediction, classification, and optimization, etc. Some literatures have been proved that the discovered dispatching rules by gene expression programming play a good performance for solving scheduling problems [3]. Therefore, this paper proposed an IGEP algorithm to realize the new dispatching rules mining as the core of the offline training part.

#### 6.2.1. Chromosome Representation and Decoding

A dispatching rule assigns the candidate jobs with highest priority firstly. It is often described as an algebraic expression in many literatures [48]. Take SPT rule as an example, SPT can be expressed as min *pt* or min (0.5 ∗ *pt* + 0.5 ∗ *pt*) algebraic expression, where, *pt* means processing time. Actually, IGEP defines the mathematical symbols and parameters symbols of the above algebraic expression as function symbol sets (FS) and terminal symbol sets (TS) [51]. This paper selects parameters {pt,nr,it} as the terminal symbols to formulate TS, where *nr* is the number of remaining unscheduled operations, and *it* is the idle time. Otherwise, five basic mathematical symbols $\left\{+,-,*,/,\sqrt{}\right\}$ are as function symbols to formulate FS.

**Figure 3:**
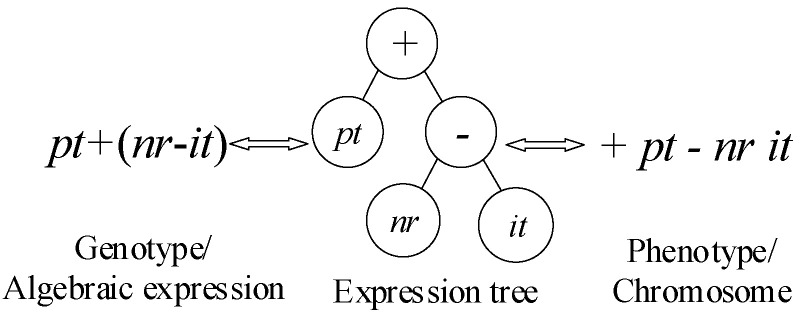
The mutual transformation of genotype and phenotype.

IGEP is a genotype/phenotype system with fixed length linear chromosomes. An expression tree enables to realize the mutual transformation of genotype and phenotype by depth-first search mode [51], as shown in Figure 3. In order to ensure that a chromosome enables to be converted into an algebraic expression successfully, one chromosome is divided into two parts: Head and Tail. The Head element can be from FS or TS, but the Tail element must be from TS. At the same time, the lengths of both Head (*h*) and Tail (*l*) are kept as fixed, and are imposed with the constraint (1) [52].

(1) l=h(m−1)

where *m* is the maximum argument in the functions.

The complexity of the encoding is directly related with the length of head. In this study, multi-gene fragments with several heads and one tail, are design to increase the diversity of algebraic expression and reduce the complexity of the encoding. Each head and the tail form one completed algebraic expression, and the mathematical symbol “+” is applied to link the algebraic expression in the same individual.

#### 6.2.2. Feasible Scheduling Scheme from Algebraic Expression

Because of job arrivals randomly, the rescheduling job set contains unprocessed job set and new job set at each rescheduling point. A matrix ϑ is selected to represent the rescheduling job set. Where *O_ij_* denotes that the *j*-th operation of job *i*. For example, when a rescheduling is triggered, the unprocessed job set = {*O*_12_ *O*_13_ *O*_23_}. The new job set = {*O*_31_ *O*_32_ *O*_33_}. The rescheduling job set matrix is defined as follow:

(2) $$\vartheta =\left(\begin{array}{ccc} O_{12}\left(2,1\right) & O_{13}\left(1,3\right) & \\ O_{23}\left(3,2\right) & & \\ O_{31}\left(5,1\right) & O_{32}\left(2,2\right) & O_{33}\left(4,2\right) \end{array}\right)$$

The algebraic expression decides the priority value of each candidate operations. The operation sequence and machine selection are known in advance. But the starting and finishing time of each operation is undetermined. Venn diagram in Pinedo [53] has verified and denoted that an active schedule contains an optimal schedule. Therefore, this research considered the active schedule only in the feasible scheduling scheme method to reduce the search space.

For example, a chromosome + *pt* – *nr nr* is given. Then the algebraic expression is denoted *pt*. There are three jobs with several operations. According to minimizing the algebraic expression *pt*, the job sequence is $\left\{O_{12},O_{13},O_{23},O_{31},O_{32},O_{23}\right\}$. In an active schedule, the machine can remain idle and the operation is assigned at the earliest starting time to be processed. Thus, the starting time and finishing time of each operation are obtained.

#### 6.2.3. Four Operators of IGEP

The IGEP algorithm generates the new dispatching rules iteratively till the stopping Criterion is meet. Four operators, including selection, mutation, recombination, and unique transposition, are utilized to update the dispatching rules in each iteration.

Selection can guarantee to keep the excellent individual into the next generation. Sometimes, it also allows the inferior individual into the next generation to extend the solution space. Tournament selection is adopted here since it endows good individuals with more survival opportunity and balances the influence of super individuals and inferior individuals [50]. A specific number of individuals are selected randomly and the best individual is cloned directly to new population with the population size times.

**Figure 4:**
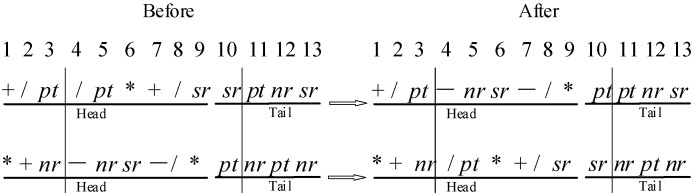
Two-point Recombination.

Recombination can keep the favorable fragments in each iteration and guarantee the convergence of the algorithm. This paper adapts two-point recombination for each candidate individual with a certain probability. As shown in Figure 4, two points are specified randomly and the middle fragments are exchanged. The position 3 and 10 are selected randomly.

**Figure 5:**
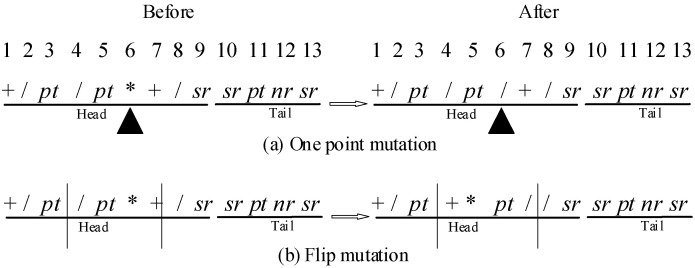
Mutation (black triangle express location of one point mutation).

Mutation aims to produce perturbations for each individual to avoid the problem of prematurity and stagnation. This paper adapts two typical mutations for each candidate individual with a certain probability. As shown in Figure 5a, the gene of one random position is replaced by other symbols in one point mutation. Flip mutation flip the fragment between two random positions, as shown in Figure 5b.

**Figure 6:**
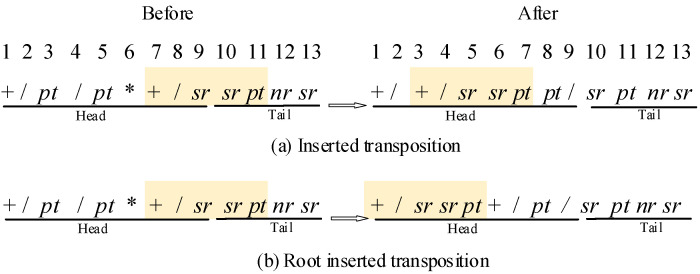
Transposition.

As all mentioned above, the length of tail makes sure the individual encodes a completed algebraic expression. At the same time, this also causes some invalid codes with excellent gene fragment in the vast majority of cases. To make full use of the information of each individual, transposition effectively activates some invalid codes with insertion and root insertion during the evolution. One random fragment is transposed into the Head of the individual before the original position in the inserted transposition, as shown in Figure 6a. Root inserted transposition transposed one random fragment with a function at the first position into the root of the individual, as shown in Figure 6b.

#### 6.2.4. Evaluation for Each Individual

Generally, a proper fitness function can efficiently guide the direction of the evolution to the optimum. It is worth noting that all the training instances come from the historical production data. It is impossible and very hard to get the optimum of these instances. Therefore, an unsupervised learning method is designed to evaluate the quality of each individual by comparing the relative merits among the population to distinguish superior and inferior solutions, as shown in Algorithm 1. The fitness function is given in Equation (3).

(3) $$F_{j}=\{\begin{array}{cc} 1/n, & \sum_{i=1}^{n}\left(\underset{\forall i}{max}f_{ij}-\underset{\forall i}{min}f_{ij}\right)<\varepsilon \\ \frac{\sum_{i=1}^{n}\frac{f_{ij}-\underset{\forall i}{min}f_{ij}}{\underset{\forall i}{max}f_{ij}-\underset{\forall i}{min}f_{ij}}}{n}, & others \end{array}$$

where *n* is the total number of training instances. $f_{ij}$ is the objective function value for the *i*-th training instance under the exact *j*-th dispatching rule, where, i=1,2,…,n, j=1,2,…,popsize. $\underset{\forall i}{max}f_{ij}$ or $\underset{\forall i}{min}f_{ij}$ means the maximum or minimum objective function value among all training instances under the exact *j*-th dispatching rule.

Obviously, the minimum objective function value $\underset{\forall i}{min}f_{ij}$ plays a crucial role on the direction of the evolution. This may cause IGEP is easy to select the local optimal solution and stop to update the solution during few iterations. To avoid the premature convergence, this paper utilizes a re-start method when the current optimal solution isn’t update in a certain iteration.
**Algorithm1.** Evaluation for each individual.1: for *j* = 1: *popsize* do 2:   $\underset{\forall i}{max}f_{ij}=0$, $\underset{\forall i}{min}f_{ij}=+\infty$ 3:   for *i* = 1: *n* do 4:      calculate *f_ij_* 5:     if $\underset{\forall i}{max}f_{ij}<f_{ij}$ then 6:       $\underset{\forall i}{max}f_{ij}=f_{ij}$ 7:     end if 8:     if $\underset{\forall i}{min}f_{ij}>f_{ij}$ then 9:       $\underset{\forall i}{min}f_{ij}=f_{ij}$ 10:     end if 11:   end for 12:  if $\sum_{i=1}^{n}\left(\underset{\forall i}{max}f_{ij}-\underset{\forall i}{min}f_{ij}\right)<\varepsilon$ then 13:    $F_{j}=1/n$ 14:  else 15:    $F_{j}=\frac{\sum_{i=1}^{n}\frac{f_{ij}-\underset{\forall i}{min}f_{ij}}{\underset{\forall i}{max}f_{ij}-\underset{\forall i}{min}f_{ij}}}{n}$ 16: end if 17: end for

#### 6.2.5. Overall Framework of the Offline Training Method

The offline training method is based on the framework of IGEP. In every training process, the rescheduling point *t* is determinate. The overall framework of the IGEP-based training method is provided in Algorithm 2.
**Algorithm2.** Overall framework of the IGEP-based offline training method.1: Inputs: Training instances I, ∀i=1,2,…,n 2: Set parameters, population size (*popsize*), maximum iteration (*maxIter*), probability of mutation (*p_m_*), probability of recombination (*p_r_*), probability of transposition (*p_tr_*), and the pre-set value for selection (*k*_*s*). 3: Initialize population randomly *P*, ∀j=1,2,…,popsize 4: Calculate each individuals *F_j_*, store the best individual into *J_best_* 5: for *iteration* = 1: *maxIter* do 6:  Apply selection to generate the new population 7:  if *p* < *p_m_* 8:   Apply recombination to generate the new population 9:  end if 10:  if *p* < *p_m_* 11:   Apply mutation to generate the new population 12:  end if 13:  if *p* < *p_m_* 14:   Apply transposition to generate the new population 15:  end if 16:  Calculate each individuals *F_j_*, store the best individual into *J_best_* 17:  iteration←iteration+1 18: end for 19: Compare *J_best_* with the dispatching rule *J_R_base_* in the rules base by testing instances. 20: if *J_best_* is better than *J_R_base_* then 21: J_*R_base*_ =J_*best*_ 22: end if

## 7. Experimental Results

The proposed dispatching rules mining and decision-making method is implemented in C++ language on a PC with Intel Core 2 Duo CPU 2.20 GHz processor and 2.00 GB RAM memory.

In this section, the detail of the dispatching rules mining and online decision-making method are provided at first. To show the superiority of the proposed method over jobs arrival randomly, this paper compares the proposed method with the discovered dispatching rule via metaheuristic algorithm. Last but not the least, the superiority of the proposed method is further validated by taking other well-known dispatching rules as comparisons. Then a sensitivity study on the shop floor parameters is conducted.

### 7.1. Data Setting and Parameter Tuning

The experimental simulation begins with a 6 × 6 static job shop problem [37]. The distribution of job arrivals process closely follows a Poisson distribution. The Poisson random variable λ is denoted as the average number of new jobs arrival per unit time. For example, λ=0.5 means that a new job with average 4 unit time arrives in shop floor. The job arrivals rate λ has four levels: 0.125, 0.25, 0.5, and 1. New jobs arrive the shop floor one by one with the quantity of new jobs from 50 to 200. This paper assumed that the processing time of each operation follows a uniform distribution between 1 and 10. Since there is few historical production data at the beginning, in order to generate some production data, online decision-making part generate scheduling scheme by calling SPT rule until the rescheduling point meets 5. In fact, the size of this value has small influence on the experiments.

The total work content (TWK) method is used to get the due date and its formulation is described as Equation (4). Two level of due date tightness factor *k*, namely loose U [2,6] and tight U [1,5] are considered. When a new job arrives, the tightness factor *k* is generated by uniform distribution.

(4) $$d_{i}=a_{i}+k\times p_{i}$$

where, $p_{i}$ denotes the total processing time of job *i*. *k* is the tightness factor.

A serial of preliminary trials was conducted and some parameters were confirmed in IGEP algorithm. The population size and number of iterations were 20 and 50 respectively. The length of Head was 8. Then the length of Tail was 9. The probability of mutation, recombination, and transposition were 0.2, 0.4 and 0.3 respectively. And the number of rules in selection was 3. The job arrivals rate, the quantity of new jobs and the due date tightness factor have 4, 4 and 2 levels separately. Each level, the problem instance is repeated independently for 10 times. Therefore, there are 4 × 4 × 2 × 10 = 320 problem instances.

### 7.2. Compared with the Discovered/Combination Rules via Metaheuristic Algorithm

To verify the effectiveness of the proposed method, this paper compares with some composite dispatching rules and the discovered rules via metaheuristic algorithm in this section. These rules are used for minimizing makespan, mean tardiness, mean flow time, maximum flowtime, and variance of flowtime. These rules include PTWINQ, RH1, RH2, RH3, GP1, GP2, GP3 [20,29,30,53]. Table 1, Table 2 and Table 3 summarizes the comparison of the discovered/combination rules with minimum makespan, total flow time and tardiness. Notes that the value of Table 1, Table 2 and Table 3 is the average value of 10 problem instance under the same scenario. Note that the first column means the new jobs number, the second column means the job arrivals rate λ, the third column means due date tightness factor *k* (L is loose, T is tight).

Table 1, Table 2 and Table 3 shows that the proposed method IGEP of this paper can obtain lower makespan, total flow time and tardiness in almost all problem instances. This means that the proposed method has discovered the proper dispatching rules at each rescheduling points. It can be seen from the results that the proposed method IGEP has a slight difference with GEP2 under the scenario of λ=1 and tight due date. ANOVA is employed to analyze all the results between IGEP and GEP2. The *p*-value is 0.980722. There was no significant difference on makespan in both methods. The main reasons may be that GEP2 was generated via the proposed method IGEP under the specific scenario. This actually proves that the proposed method IGEP can discover an efficient rule for the specific scenario. However, the problem condition in intelligent job shop always keeps changing. Therefore, the proposed method IGEP has superior performance by discovering the dispatching rules based on the current problem condition.

At the same time, GEP2 is superior than the PTWINQ, RH1, RH2, RH3, GP1, GP2, GP3 and GEP1 in all the problem instances when the objective is minimum makespan. But GEP1 is superior than the PTWINQ, RH1, RH2, RH3, GP1, GP2, GP3 and GEP2 in all the problem instances when the objective is minimum total flow time and tardiness. This shows that the tiny confliction of three objectives: makespan, total flow time and tardiness is obvious. Moreover, Table 2, Table 3 and Table 4 shows that PH3 plays a best performance among all the mention rules excluded the IGEP rules. The makespan, total flow time, and tardiness of RH3 in all the problem instances are better than other 6 rules: PTWINQ, RH1, RH2, GP1, GP2 and GP3. This shows that the objective of makespan, total flow time and tardiness are certain consistent and correlation.

**Figure 7:**
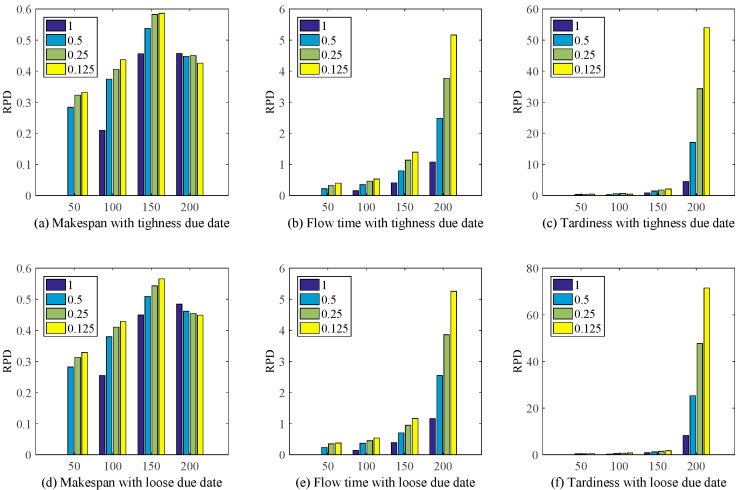
RPD for RH3 and IGEP under different scenario.

In order to illustrated the effectiveness and improvement of the proposed method IGEP, Meanwhile, the relative percentage deviation (RPD) is utilized to evaluate the results of experiments. It can be calculated as Equation (5). Figure 7 gives RPD between the RH3 rules and the proposed method IGEP.

(5) $$RPD=\frac{F_{RH3}-F_{GEP}}{F_{GEP}}\times 100\%\;$$

where, $F_{RH3}$ and $F_{GEP}$ are the objective value via RH3 rules and the proposed method IGEP.

Figure 7 shows the proposed method IGEP can remarkably improve the schedule performance. At the same time, when λ = 0.125, the improvement performance on the whole is most obvious. Though RH3 plays a good performance for makespan. But Figure 7b,c,e,f show that the RH3 deteriorated drastically when the objective function is Flow time or Tardiness. Meanwhile, with the increase of the number of jobs arrival, the performance improvement of IGEP is highly significant.

To summary, the proposed method IGEP can discover the efficient dispatching rules under the specific scenario at each rescheduling point. This makes sure that the intelligent job shop can keep stability and efficient for a long time. Meanwhile, according to the decision maker’s expectation, the proposed method IGEP can modify its objective to obtain the good performance.

### 7.3. Compared with Other Well-Known Dispatching Rules

In order to further confirm the superiority of the proposed method IGEP, this paper compare the proposed method IGEP with other nine well-known dispatching rules, including SPT, LPT, SRM, LRM, LOPR, MOPR, SWKR, MWKR, WINQ [20,29,30].

Table 4, Table 5 and Table 6 summarizes the comparison of the well-known dispatching rules with minimum makespan, total flow time and tardiness. Notes that the value of Table 4, Table 5 and Table 6 is the average value of 10 problem instance under the same scenario. Note that the first column means the new jobs number, the second column means the job arrivals rate λ, the third column means due date tightness factor *k* (L is loose, T is tight).

Table 4, Table 5 and Table 6 indicated that the proposed method IGEP of this paper produced the lowest makespan, total flow time and tardiness in all problem instances. Specifically, the proposed method IGEP is significantly superior to the well-known dispatching rules for all problem instances. Meanwhile, MOPR has excellent performance than other well-known dispatching rules, excluded the proposed method IGEP.

In order to illustrated the effectiveness and improvement of the proposed method IGEP, Meanwhile, the relative percentage deviation (RPD), as shown in Equation (6), is also utilized to evaluate the performance between MOPR rules and the proposed method IGEP.

(6) $$RPD=\frac{F_{MOPR}-F_{GEP}}{F_{GEP}}\times 100\%\;$$

where, $F_{MOPR}\;$ and $F_{GEP}$ are the objective value via the MOPR rules and the proposed method IGEP.

**Figure 8:**
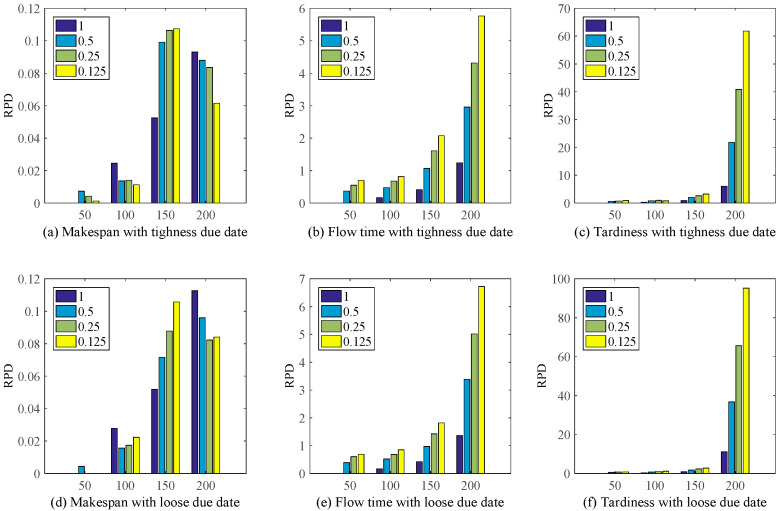
RPD for MOPR and IGEP under different scenario.

Figure 8 shows the proposed method IGEP can remarkably improve the schedule performance. At the same time, when λ = 0.125, the improvement performance on the whole is most obvious. Though MOPR plays a good performance for makespan. But Figure 8b,c,e,f show that the MOPR deteriorated drastically when the objective function is Flow time or Tardiness. Meanwhile, with the increase of the number of jobs arrival, the performance improvement of IGEP is highly significant. This conclusion is same with Figure 7. This illustrates that the single dispatching rules sometimes has good performance on one scenario, such as the makespan objective function. But the proposed method IGEP can discover the efficient dispatching rules based on the current environment or scenario.

In summary, to make full use of the historical production data, the proposed method IGEP has superior performance for the intelligent job shop. Due to the difference among the scenario and the preference of decision maker, different dispatching rules show a big difference for the intelligent job shop. However, the proposed method IGEP with strong self-adaptive can discover the current efficient dispatching rules and the decision makers’ expectation anytime with the real production condition.

### 7.4. Sensitivity Study on the Experimental Parameters

**Figure 9:**
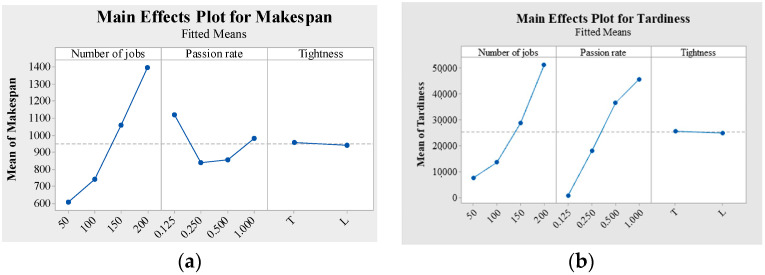

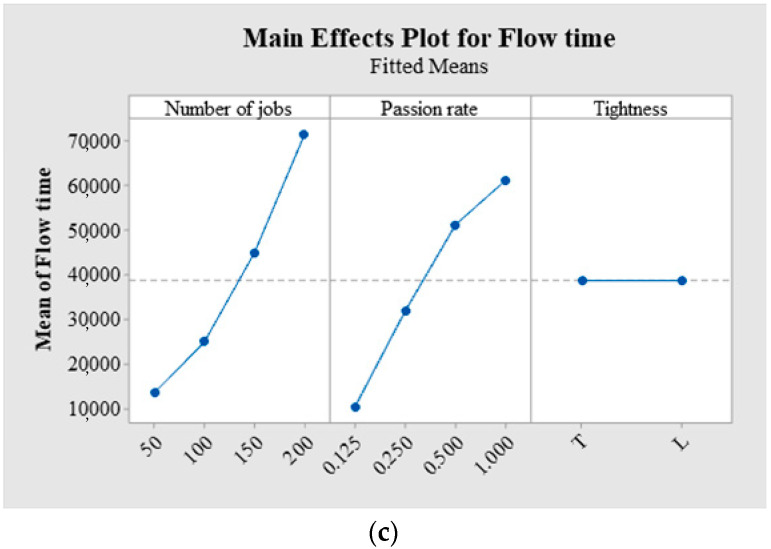
Factorial plot for different objective functions. (**a**) Main effects Plot for Makespan. (**b**) Main effects Plot for Tardiness. (**c**) Main effects Plot for Flow time.

The number of jobs, Passion rate and tightness factor plays an important role in affecting the performance of intelligent shop floor. The number of jobs mainly means the load level of shop floor during a period of time. Passion rate and tightness factor are the critical parameters to decide the tightness level of jobs arrival and due date. Therefore, this paper utilizes the multi factorial analysis of variance to test the parameter sensitivity. Figure 9 provides the factorial plot for different objective functions.

Table 7 show the ANOVA of different objective functions. In this study, effects are considered significant if the *p* value is less than 0.05.

Table 7 shows that Passion rate and number of jobs are significant for all the performance measures since the *p* value is less than 0.05. But Tightness is not significant for all the performance measures since the *p* value is larger than 0.05. In detail, makespan, flow time and tardiness increase with the increase of the number of jobs as shown in Figure 9. There is a valley in Figure 9a when the Passion rate equals 0.25 and 0.5. In Figure 9b,c, flow time and tardiness increase with the density of Passion rate. Hence, for the performance measure of makespan, total flow time and tardiness, the Passion rate and the number of jobs factor have a statistically significant impact on the performance measure.

## 8. Conclusions

This paper proposes a new scheduling framework to achieve real-time decision making for the intelligent shop floor with random jobs arrival. At each rescheduling point, the appropriate dispatching rules are discovered by IGEP to assign the unprocessed operation on an available machine and generate the scheduling scheme in real time. Numerical experiments under different production scenario are conducted to verify the effectiveness and superiority of the proposed framework.

The main conclusions are as follows.

(1) The real-time decision-making ban be achieved by calling the dispatching rules at each rescheduling point. It can be quite satisfying to reach up to the requirements of the real application.
(2) Due to historical production data-driven, the discovered dispatching rules at each rescheduling point have significant superiority in the current specific production scenario. These rules with low computational requirement and easy implementation in real intelligent job shop scheduling problem.
(3) The IGEP algorithm owns the merits of artificial intelligence via high self-study and self- adaptability, and also has the advantage of exploring the potential and appropriate scheduling knowledge from the historical production data.

In future work, more uncertainty and dynamic scenario of shopfloors, such as machine breakdowns and processing time variations will be considered. More scheduling efficient objectives like energy consumption and scheduling stability objectives are also worthy to be studied.

## Figures and Tables

**Table 1:** Comparison of discovered/combination rules with minimum makespan.

			PTWINQ	RH1	RH2	RH3	GP1	GP2	GP3	GEP1	GEP2	IGEP
50	1	T	1074.6	1074.6	1074.6	1074.6	1074.6	1074.6	1074.6	1074.6	**1074.6**	**1074.6**
		L	1039.4	1039.4	1039.4	1039.4	1039.4	1039.4	1039.4	1039.4	**1039.4**	**1039.4**
	0.5	T	991.8	1003.8	1189.5	612.9	1091.9	1098.5	1057.3	528.7	**504.5**	506.4
		L	1015	997	1261.7	558.4	1098.6	1125.7	1070.9	471.3	447.2	**444.9**
	0.25	T	1029.5	1062	1314.4	591.2	1164.5	1207.5	1057.9	446.5	**401.7**	406.1
		L	1020.5	1047.7	1286.2	583.6	1174.3	1177.8	1045.4	436.1	**401.4**	402.6
	0.125	T	1038.1	1030	1249.5	727.6	1190.4	1191.9	920.6	530.4	501.7	**499.4**
		L	1041.8	1035.5	1248.3	708.4	1151.4	1193.6	943.4	503.5	**475**	477.3
100	1	T	1888.5	1995.6	2578	869.5	2166.3	2230.4	2051.6	722.9	677.6	**677**
		L	1873.9	1973.2	2516	848.6	2164.6	2239.6	2035.5	704.9	**662.2**	**661.9**
	0.5	T	1915.5	1946.7	2450.5	948.1	2142.8	2231.5	2002.3	734.3	**689.8**	**689.8**
		L	2026.8	2070.8	2613.6	952.6	2272	2296.4	2018.9	739.5	**690.5**	**690.5**
	0.25	T	1974.3	1980.1	2502.6	1073.7	2213.6	2280.5	1881.6	749.8	**691.2**	698.1
		L	1918.1	1993.1	2508.3	1032.4	2190.5	2269.1	1872	733.2	677.8	683.9
	0.125	T	1984.3	1981.9	2368.4	1336.4	2231.7	2242.5	1673.3	947.9	924.2	**923.2**
		L	2007.7	1962.8	2388.5	1309.2	2198.7	2304.7	1662.7	923.5	903	**895.8**
150	1	T	2821.4	3024.4	3833.5	1264.4	3198	3332.1	3009.3	1015	956.4	**955.5**
		L	2863.3	2991.1	3830.9	1239.5	3268.5	3387.4	2990.6	1014.8	944.7	**944.2**
	0.5	T	2825.6	2937.2	3727.9	1382.6	3224.7	3347.4	2897.7	1037.7	**981.5**	983.4
		L	2991	3034.2	3894.1	1382.5	3357.4	3431.5	2951	1042	**978.3**	980.2
	0.25	T	2856.7	2964.5	3727.4	1555.2	3273	3383.7	2674.8	1049.4	**972**	982.8
		L	2861.2	2977.6	3717.5	1518	3299.5	3385.2	2750.8	1046.5	**971.8**	983.8
	0.125	T	2914.7	2975.3	3528.2	1923.4	3282.1	3327.3	2393.8	1350.8	1327.5	**1326**
		L	2941.7	2920.5	3571.9	1904.3	3281.1	3396.5	2390.1	1328	1310.1	**1309.4**
200	1	T	3819.6	4108.3	5183.7	1669.9	4376.8	4520.4	4003.2	1337.1	1254.1	**1254**
		L	3906.6	4063.7	5253.1	1666.3	4393.6	4567.6	3981.3	1337.4	**1253.3**	1253.6
	0.5	T	3827.8	4020.1	5076.6	1827.6	4350.6	4514.1	3837.1	1347.4	**1267.9**	1271.9
		L	3944.2	4110.4	5244.9	1820	4464.3	4578.1	3870.7	1357.9	**1271.2**	1273.8
	0.25	T	3815.4	4039.7	4992.2	2039.3	4382.8	4501.7	3545.4	1362.3	**1264.8**	1285.6
		L	3854	3904.2	4994.3	2011.4	4364.4	4503.2	3567	1356.3	**1269.3**	1284.6
	0.125	T	3874.9	3912.7	4669.2	2558	4352	4436.4	3090.7	1815.4	1797	**1794.1**
		L	3880.2	3869.2	4707.4	2517.7	4364.1	4466.2	3081	1757.8	1738.9	**1737.8**

**Table 2:** Comparison of discovered/combination rules with minimum total flow time.

			PTWINQ	RH1	RH2	RH3	GP1	GP2	GP3	GEP1	GEP2	IGEP
50	1	T	27,224.5	27,224.5	27,224.5	27,224.5	27,224.5	27,224.5	27,224.5	27,224.5	27,224.5	**27,224.5**
		L	25,233.7	25,233.7	25,233.7	25,233.7	25,233.7	25,233.7	25,233.7	25,233.7	25,233.7	**25,233.7**
	0.5	T	24,441.7	33,230.5	31,892.6	16,077.3	24,437.7	24,408.1	24,237.2	14,160.9	15,255	**13,894.6**
		L	24,038.9	33,058	33,393.1	14,920.6	24,106.5	24,511.4	24,012.4	12,955.3	14,406.6	**13,082**
	0.25	T	24,248.2	35,934.2	34,661.6	13,897.5	25,648.2	26,515.6	21,726.3	10,606.5	12,529.8	**9864**
		L	23,934.2	34,910.8	34,082.3	13,455	25,639.6	25,617.2	20,995.4	10,123.4	12,236.7	**9703.6**
	0.125	T	19,073.2	26,788	25,558.8	11,149.4	21,777.6	22,120.7	13,042.7	5917.2	6449.3	**5350.7**
		L	18,908.1	26,969.3	25,735.8	11,316.5	20,643.1	21,845.6	13,399.1	5845.1	6531.1	**5244.2**
100	1	T	92,786.7	142,077.1	139,488.9	45,350	93,993	95,471.1	93,012.4	38,099	48,963.9	**37,113.7**
		L	90,151.3	138,283.1	135,782.5	44,171.3	93,240.4	94,048.5	90,654.8	36,340.8	47,756.7	**35,788.6**
	0.5	T	89,267	135,859.2	130,829.4	44,163.6	91,083.1	94,804.6	83,381.6	34,250.1	44,867.9	**32,665.5**
		L	95,275	143,177.3	138,559	44,168.1	97,096.8	96,712	84,967.9	34,963.8	46,462.9	**32,290**
	0.25	T	84,085.8	126,218.5	122,818.6	40,073.7	89,828.2	94,065.8	66,485.3	24,934	37,684.1	**22,313.7**
		L	81,439	125,729.7	122,325.1	38,315.6	87,727.3	91,408.1	65,143.5	24,726.3	36,684.1	**22,505.1**
	0.125	T	63,625.4	93,736.6	87,527.4	30,413.3	73,309.2	77,736.4	35,468.9	10,280.3	10,698.3	**8715.8**
		L	64,998.5	95,745.8	90,721.2	31,192.7	72,116.6	79,313.4	36,350.3	10,321.3	10,883	**8793**
150	1	T	204,569.7	322,195.8	315,526.2	92,377.6	206,362.6	213,759.7	200,236	75,800.3	103,499.4	**69,631.3**
		L	204,407.2	322,299.4	309,511.3	90,167.5	209,066.1	213,110.9	198,102.8	73,973.8	103,419.6	**66,854.4**
	0.5	T	195,613.8	309,206.2	294,512.3	88,714.8	204,084.1	211,248	175,535.8	66,589.7	94,761.6	**60,597.9**
		L	207,092.5	317,950.4	310,949.7	88,995.7	213,978.2	216,953.7	179,185.2	66,905.6	97,213.2	**61,358.7**
	0.25	T	175,988.4	282,124.5	269,352.2	78,917.2	193,117.1	202,920.4	130,847.7	44,178.2	75,130.1	**36,838.1**
		L	178,088.6	284,430.9	271,702.6	77,193.9	194,400	202,939.9	136,853.4	44,875.3	76,400.4	**39,648.5**
	0.125	T	132,407.8	209,223.4	190,462.7	58,954.6	155,456.9	167,921.8	66,599.8	14,655.2	15,065.9	**12,359.3**
		L	136,123.5	208,675.1	194,796.2	59,607.5	157,480.9	169,850.3	68,239.5	14,560.2	15,481.2	**12,261.6**
200	1	T	373,429.3	596,443.1	577,584.4	159,719	385,104.6	393,346.8	356,041.8	126,889.4	185,066.9	**114,124**
		L	377,988.8	593,816.6	577,838	157,174.4	381,946.4	394,165.1	352,694.6	125,705.9	185,770	**114,017.6**
	0.5	T	353,438.3	574,122.2	540,786	151,360.7	372,818.8	383,075.1	306,174.1	108,902.3	165,472	**98,491.1**
		L	360,244	579,227.6	557,606.8	150,348.6	376,191.6	382,466.2	307,384.6	109,085.1	167,952	**97,736**
	0.25	T	311,517.4	514,774	477,247.8	131,825.9	341,554.7	355,814.2	225,592.7	67,327.8	127,236.1	**54,964.3**
		L	314,377.7	499,506.9	484,014.9	130,140.9	342,117.3	357,962.9	229,184.5	69,333.7	129,773.9	**60,080.3**
	0.125	T	230,656.1	363,731.4	332,335.7	96,621.6	268,951.2	295,484.4	104,743.6	18,691.1	18,861.6	**15,679.6**
		L	232,664.9	366,069.3	338,412.4	98,136.1	274,425.3	295,171.2	107,654.4	18,880.4	19,505.2	**15,685.8**

**Table 3:** Comparison of discovered/combination rules with minimum tardiness.

			PTWINQ	RH1	RH2	RH3	GP1	GP2	GP3	GEP1	GEP2	IGEP
50	1	T	20,533.6	20,533.6	20,533.6	20,533.6	20,533.6	20,533.6	20,533.6	20,533.6	20,533.6	**20,533.6**
		L	18,710.5	18,710.5	18,710.5	18,710.5	18,710.5	18,710.5	18,710.5	18,710.5	18,710.5	**18,710.5**
	0.5	T	17,882.5	26,644.3	25,310.1	9533.2	17,911.9	17,874.4	17,754.5	7689.9	8679.2	**7434.4**
		L	17,482.9	26,452.7	26,801.5	8352.3	17,609.2	18,017.9	17,534.8	6436.1	7808.2	**6377.4**
	0.25	T	17,337.2	28,858.6	27,590.4	6917.2	18,812.7	19,679.1	14,975.5	3753.2	5603.6	**3635.5**
		L	17,187.2	27,987.2	27,167	6644	18,949.1	18,904.8	14,346.3	3504.2	5440.5	**3608.9**
	0.125	T	12,833.6	19,961.7	18,762.6	4503.1	15,704.9	15,974.6	6970.9	835.6	1626.6	**811.2**
		L	12,362.1	19,809.1	18,636.3	4377	14,201.8	15,296.7	7050	530.7	1321.8	**470.6**
100	1	T	79,547.7	128,803.7	126,221.3	32,120	80,810.3	82,291	79,858.4	24,940.7	35,690.5	**23,044.5**
		L	76,822.6	124,931.2	122,433.6	30,842	79,979.1	80,785.9	77,443.6	23,095.5	34,404.8	**22,604.2**
	0.5	T	76,163	122,594.6	117,571.3	30,955.5	78,106.8	81,810.2	70,392.1	21,133.9	31,603.3	**19,362.3**
		L	81,948.3	129,720.2	125,115.5	30,751.9	83,928.6	83,547.5	71,758	21,623.9	33,005.8	**20,694.6**
	0.25	T	70,932.2	112,555.4	109,159.5	26,505.9	76,852.3	81,090.7	53,439.8	11,500.8	24,141.3	**10,601.7**
		L	68,517.3	112,369.9	108,973.6	25,068.4	74,979.5	78,640.6	52,317.6	11,676.5	23,424.9	**11228.8**
	0.125	T	51,564	80,325.1	74,146	17,188	61,491.2	65,734.2	23,418.3	1066.1	2090.7	**948.9**
		L	52,603.6	81,934.9	76,971	17,602.5	59,855.8	66,903.7	23,948.2	776.8	1756.6	**668.9**
150	1	T	184,836.2	302,350.3	295,686.5	72,575.5	186,770.6	194,170.9	180,621.2	56,077	83,653.9	**50,811.9**
		L	184,455.3	302,260.6	289,475.5	70,151.3	189,269.1	193,326.6	178,355	54,041.5	83,380.8	**50,196.5**
	0.5	T	175,773.6	289,108.4	274,421	68,673.5	184,443.1	191,609.7	155,848.8	46,641.3	74,663.8	**41,503**
		L	187,060.7	297,681.1	290,694	68,767.3	194,213	197,227	159,304.7	46,753.9	76,943.9	**41,585.8**
	0.25	T	156,630	261,858.2	249,089.9	58,746.2	174,043.8	183,810.7	111,482.6	24,141.7	54,934.2	**20,945.8**
		L	158,703.8	264,323	251,603	57,198.6	175,279.7	183,816.2	117,488.8	25,081.6	56,334.3	**22,907.7**
	0.125	T	114,495.3	189,187	170,456.4	39,104.4	137,869.4	150,100.2	48,405	1242.4	2516.3	**1104.6**
		L	117,653.7	188,004.1	174,185.9	39,157.2	139,051.3	151,330.4	49,561.2	936.1	2440.5	**804.1**
200	1	T	346,471.9	569,314.9	550,462	132,634.2	358,325.6	366,639.1	329,214.1	99,885.7	157,938.7	**89,508.2**
		L	350,901.6	566,553.7	550,578.1	129,934.1	355,080.9	367,313.8	325,800.2	98,549.9	158,507.1	**90,372.7**
	0.5	T	326,877.3	547,187.9	513,858.2	124,482.9	346,529.3	356,769.8	279,805.5	82,117.4	138,537.7	**84,899.1**
		L	333,496.8	552,099.9	530,492.7	123,261.8	349,800.2	356,104.5	280,771.5	82,075	140,824.3	**70,765.6**
	0.25	T	285,586.4	487,559.1	450,036.9	104,706.3	316,013.3	330,203.7	199,487.8	40,342.8	100,107.7	**33,508.7**
		L	288,698.1	472,782.5	457,298.8	103,529.1	316,806	332,611.2	203,469.5	42,923.5	103,114.8	**37,722.4**
	0.125	T	206,839.1	336,861.7	305,496.1	69,938.1	245,456.5	271,701	80,231.7	1395.9	2704.8	**1272.3**
		L	208,445.2	338,842.7	311,246.5	71,130.2	250,289.6	270,860.5	82,989.5	1138.1	2717.4	**981.1**

**Table 4:** Comparison of discovered/combination rules with minimum makespan.

			SPT	LPT	SRM	LRM	LOPR	MOPR	SWKR	MWKR	WINQ	IGEP
50	1	T	1074.6	1074.6	1074.6	1074.6	1074.6	1074.6	1074.6	1074.6	1074.6	**1074.6**
		L	1039.4	1039.4	1039.4	1039.4	1039.4	1039.4	1039.4	1039.4	1039.4	**1039.4**
	0.5	T	969.6	1001.4	1199.1	553.9	1219.1	518.8	1174.9	551.7	1015.4	**506.4**
		L	991.8	985.7	1215.4	506.9	1222.5	457.3	1211.7	496.4	1047.7	**444.9**
	0.25	T	1011.6	1050.6	1331.9	480.7	1303.3	427.4	1303.7	469.9	1091	**406.1**
		L	978	1019	1295.1	466.7	1333.2	423.5	1282.2	472.8	1054	**402.6**
	0.125	T	1002.5	1049.4	1320.3	584.1	1328	545.9	1283.2	575.7	1081.8	**499.4**
		L	999.8	1054.1	1297	563.1	1332	531.1	1303.6	555.9	1063.9	**477.3**
100	1	T	1863.7	1932.8	2473.5	755.1	2496.8	681.9	2448.5	745.7	1951.2	**677**
		L	1857.9	1894.8	2474.9	742.6	2475.8	664.8	2459.8	746.8	1985.3	**661.9**
	0.5	T	1847.4	1915.7	2462.8	773.7	2498.2	699.2	2464.6	781	1971.7	**689.8**
		L	1953.1	1976.7	2555.9	795.9	2546.3	701.3	2513.5	787.4	2089.2	**690.5**
	0.25	T	1878.8	1981	2523.1	865.5	2542.6	767.3	2491.2	845.8	2051.7	**698.1**
		L	1858.8	1930.1	2455.2	823.5	2529.7	732.9	2450.1	818.7	1993.9	**683.9**
	0.125	T	1886.9	1970.9	2541.4	1071.7	2554.2	1004.4	2523.2	1057.1	2049.6	**923.2**
		L	1871.3	1994.2	2495.4	1047.7	2580.3	981.8	2520.8	1035.8	2044.7	**895.8**
150	1	T	2731.2	2895.6	3684	1064	3695.1	959.5	3678.1	1053.2	2918.9	**955.5**
		L	2797.4	2858.2	3746.5	1054.2	3728.3	943.7	3710.3	1048.8	2976.6	**944.2**
	0.5	T	2743.3	2908.1	3713.4	1100.6	3771.5	997.2	3685.2	1100.2	2970.5	**983.4**
		L	2835.8	2921.5	3769.1	1120.2	3821.7	997.3	3758.4	1101.6	3029.6	**980.2**
	0.25	T	2794.4	2928.5	3735.6	1229	3774.1	1087.4	3706.5	1217.3	3018.1	**982.8**
		L	2758.3	2889.6	3682.6	1190.3	3817.6	1070.2	3691	1178.9	3002	**983.8**
	0.125	T	2795.3	2894.7	3752.2	1539.3	3797.2	1436.8	3720	1520.8	2993.1	**1326**
		L	2772.4	2931.1	3713.2	1517.1	3813.1	1417.1	3754.4	1503.6	3028.9	**1309.4**
200	1	T	3704.6	3900.8	4971.6	1391	5043.6	1255.5	4996.3	1400.7	3949.7	**1254**
		L	3781.5	3876.7	5063.7	1385	5077.2	1252.2	5034.3	1381.1	4035.8	**1253.6**
	0.5	T	3687.2	3918	4985.2	1430.3	5046.5	1286.2	4959.5	1433.2	4013.8	**1271.9**
		L	3788.5	3887.2	5011	1454.3	5092.7	1302.3	5038.9	1434.7	4033.7	**1273.8**
	0.25	T	3728.4	3872	5017.6	1587.8	5052.7	1423.7	4950.7	1577.8	4015.8	**1285.6**
		L	3693.2	3841	4919.8	1573.3	5091	1420.5	4949.5	1532.6	3995.9	**1284.6**
	0.125	T	3711.3	3862.8	4996.6	2033.7	5047.3	1904.5	4971	2015.5	3969.5	**1794.1**
		L	3671.6	3886.7	4946.4	2005.1	5060	1884	4993.7	1988.9	4002.2	**1737.8**

**Table 5:** Comparison of discovered/combination rules with minimum total flow time.

			SPT	LPT	SRM	LRM	LOPR	MOPR	SWKR	MWKR	WINQ	IGEP
50	1	T	27,224.5	27,224.5	27,224.5	27,224.5	27,224.5	27,224.5	27,224.5	27,224.5	27,224.5	**27,224.5**
		L	25,233.7	25,233.7	25,233.7	25,233.7	25,233.7	25,233.7	25,233.7	25,233.7	25,233.7	**25,233.7**
	0.5	T	25,367	28,762.7	26,538.3	18,086.4	29,179.1	16,208.7	26,031.2	17,914.4	25,199	**13,894.6**
		L	25,481	29,491.1	26,230.8	17,267.4	28,659.6	15,307.2	26,031.9	17,195.1	25,985.2	**13,082**
	0.25	T	25,558.2	30,896.9	27,880.9	16,336.8	30,395.1	13,945.9	27,049.2	16,315.3	26,523.5	**9864**
		L	24,473.8	29,764.3	26,547.6	15,904.2	30,748.6	13,842.9	26,569.6	16,447.1	26,038.6	**9703.6**
	0.125	T	20,097.5	25,653.3	22,624.7	15,439.8	26,027.5	11,986.6	21,504.5	15,058	20,987.2	**5350.7**
		L	19,035.8	24,948.3	21,851.3	15,444.4	26,106.1	12,425.4	21,948.3	14,973	20,456.2	**5244.2**
100	1	T	99,112.4	117,741.8	104,772.2	57,637	118,810.7	50,844.2	102,906.1	58,063.9	98,395.6	**37,113.7**
		L	96,182.5	114,172.1	104,160.6	56,945.5	116,116.2	49,804.5	102,523.8	58,117.7	98,511	**35,788.6**
	0.5	T	94,013.1	112,281	101,616.1	56,170.7	116,604.4	48,142.4	100,827.7	57,115.4	95,243.8	**32,665.5**
		L	99,156.3	118,706.1	106,127.5	58,120.2	117,269.8	49,244.2	104,129.4	58,531.7	103,462.2	**32,290**
	0.25	T	86,467	110,893	97,038.2	55,704.4	109,885.7	46,081.8	95,112.1	55,241.5	92,568.6	**22,313.7**
		L	85,136.6	106,444.7	92,318.7	52,635.2	110,154.2	44,461.5	92,128	53,240.5	89,722.2	**22,505.1**
	0.125	T	64,887.4	85,448.6	76,893.4	51,505.8	91,053.9	34,471.9	75,216.1	49,034.4	70,185.7	**8715.8**
		L	64,722.4	87,397.7	75,101.9	52,619	93,653.2	38,588.9	76,328.2	51,683.3	71,398.2	**8793**
150	1	T	216,121.4	263,288.9	232,508.7	122,514.2	265,876.6	107,791	230,420	123,236.2	219,096.4	**69,631.3**
		L	216,246	259,781.8	235,140.5	121,898.5	264,146.6	107,478	231,549.8	123,811.6	220,239.6	**66,854.4**
	0.5	T	206,342.8	253,886.7	226,852.7	118,218.2	259,345.4	101,493.6	223,336.1	120,346.6	213,556.8	**60,597.9**
		L	216,769.4	261,532.1	231,611.3	121,830.8	263,271.5	103,520.1	229,419.5	122,883	223,058.3	**61,358.7**
	0.25	T	188,564.8	239,162.3	210,027.3	115,352	240,296.3	96,130.6	205,856.2	117,168.9	198,005.1	**36,838.1**
		L	187,739	239,132.6	204,412.4	114,100.3	245,338.8	96,287.6	205,583.8	114,638.5	199,583.3	**39,648.5**
	0.125	T	137,160.3	185,599	164,794.7	108,463.6	196,893	65,683.1	161,226.8	103,360.1	146,186.3	**12,359.3**
		L	136,049.9	188,810.2	161,983.1	109,433.6	200,729.8	73,804.9	163,627.9	107,428.1	150,689.4	**12,261.6**
200	1	T	394,656.6	480,846.3	424,225.6	220,485.5	487,352.8	193,205	425,035.7	224,262.4	401,391	**114,124**
		L	398,032.7	477,883	432,521.8	218,964.7	488,024.9	193,208.9	425,772.1	222,543.4	406,202.3	**114,017.6**
	0.5	T	370,484.8	464,258.9	406,416.6	206,974.8	467,858.6	178,291	401,960.7	211,480.5	387,306.9	**98,491.1**
		L	381,712.6	465,508.5	406,692.7	213,512	470,242	180,820.4	404,886.6	213,256.7	388,877.7	**97,736**
	0.25	T	334,137	420,566.6	371,511.7	199,130.3	426,609.7	169,031.3	362,286.6	202,354.8	349,292.9	**54,964.3**
		L	331,423.9	424,995.8	359,986.7	201,033.5	436,141.4	169,503.1	363,879.9	198,520.7	351,902.1	**60,080.3**
	0.125	T	240,061.4	324,089.6	287,391.1	191,035.5	345,463	106,100.8	282,653.3	181,933.2	256,071.8	**15,679.6**
		L	235,147.8	326,479	282,299.4	191,882.2	352,169.1	121,131.5	284,588.7	185,692.6	258,831.2	**15,685.8**

**Table 6:** Comparison of discovered/combination rules with minimum tardiness.

			SPT	LPT	SRM	LRM	LOPR	MOPR	SWKR	MWKR	WINQ	IGEP
50	1	T	20,533.6	20,533.6	20,533.6	20,533.6	20,533.6	20,533.6	20,533.6	20,533.6	20,533.6	**20,533.6**
		L	18,710.5	18,710.5	18,710.5	18,710.5	18,710.5	18,710.5	18,710.5	18,710.5	18,710.5	**18,710.5**
	0.5	T	18,791.8	22,222.4	19,998.7	11,500.2	22,621.4	9623	19,488.4	11,328.2	18,625.3	**7434.4**
		L	18,897.7	22,911.8	19,700.5	10,662.1	22,102.2	8701.9	19,515.8	10,589.8	19,408.9	**6377.4**
	0.25	T	18,612.2	23,929.8	21,046.7	9268.3	23,371.8	6908.5	20,164.6	9254.7	19,549.4	**3635.5**
		L	17,680.9	22,954.2	19,823.6	8983.8	23,892.3	6946.2	19,828.7	9539	19,237.9	**3608.9**
	0.125	T	13,771.6	19,182.2	16,405.7	8797.6	19,257.4	5696.6	15,265.5	8598.1	14,656.7	**811.2**
		L	12,351.8	18,305.6	15,300.1	8435.7	19,025.4	5703.4	15,313.9	8119.9	13,732.3	**470.6**
100	1	T	85,844.4	104,481.2	91,578.4	44,363.6	105,601.1	37,570.8	89,706.9	44,790.5	85,134.6	**23,044.5**
		L	82,833.5	100,841.9	90,895.4	43,593.6	102,801.2	36,452.6	89,243.1	44,765.8	85,162.8	**22,604.2**
	0.5	T	80,800.5	99,100	88,597.8	42,906.1	103,385.5	34,877.8	87,842.8	43,850.8	82,054.3	**19,362.3**
		L	85,740.8	105,266.7	92,916.3	44,663.1	103,868.4	35,787.1	90,925.3	45,074.6	90,062.8	**20,694.6**
	0.25	T	73,122.7	97,448.3	84,033.3	42,041.3	96,274.9	32,418.7	82,062.5	41,578.4	79,159.5	**10,601.7**
		L	72,100	93,278.9	79,504	39,275.4	96,861.7	31,101.7	79,266.6	39,880.7	76,718.4	**11,228.8**
	0.125	T	52,506.7	72,728	64,783.9	38,190.3	77,698.6	21,644.9	63,114	36,023.6	57,827.4	**948.9**
		L	51,984.6	74,491.2	62,725.9	38,895.5	79,921.8	25,293.8	63,786.2	38,263.8	58,575.7	**668.9**
150	1	T	196,298.8	243,479.5	212,857.6	102,668.7	246,094.9	87,945.5	210,753.6	103,390.7	199,277.9	**50,811.9**
		L	196,224.3	239,786.7	215,285.8	101,859.7	244,144.7	87,439.2	211,685.8	103,772.8	200,214.3	**50,196.5**
	0.5	T	186,346.6	233,907.5	207,151	98,120.4	239,293.3	81,395.8	203,686.5	100,248.8	193,566.5	**41,503**
		L	196,632.7	241,344.8	211,832.1	101,561.5	243,057.9	83,250.8	209,628.8	102,613.7	202,913.6	**41,585.8**
	0.25	T	168,897.3	219,248.3	190,863.5	95,085.7	220,082.3	75,864.3	186,642.5	96,902.6	178,296.3	**20,945.8**
		L	168,103	219,350.8	185,165.3	93,992.4	225,298.2	76,179.7	186,302.3	94,530.6	180,035.4	**22,907.7**
	0.125	T	118,781.8	166,656.9	146,718.6	88,474.6	176,912.8	46,245.1	143,154.5	83,740.2	127,841	**1104.6**
		L	117,061.5	169,528.1	143,473.7	88,792.3	180,138.3	53,607.2	144,918.8	87,192.8	131,600.2	**804.1**
200	1	T	367,566.8	453,777.9	397,394.9	193,357.3	460,288.4	166,076.8	398,191.1	197,134.2	374,303.4	**89,508.2**
		L	370,802.5	450,657.1	405,571.5	191,701.8	460,798.9	165,946	398,818.5	195,280.5	378,982	**90,372.7**
	0.5	T	343,700.2	437,474.2	380,128.6	180,040.5	440,970	151,356.7	375,668.4	184,546.2	360,535.8	**84,899.1**
		L	354,788.9	438,528.3	380,249	186,384.3	443,170	153,692.7	378,442.5	186,129	361,946.7	**70,765.6**
	0.25	T	307,700.7	393,793.3	345,820.5	171,915.4	399,447.1	141,816.4	336,503.8	175,139.9	322,808.9	**33,508.7**
		L	305,354.9	398,724	334,524.6	174,309.1	409,484.3	142,778.7	338,333.8	171,796.3	325,958.1	**37,722.4**
	0.125	T	215,481.6	298,779.2	263,354.1	164,201.6	318,649.5	79,942.9	258,465.7	155,595.8	231,508.3	**1272.3**
		L	210,275.5	301,127.5	257,979.3	164,650.4	325,022	94,324.4	260,090.1	158,884.6	233,877.2	**981.1**

**Table 7:** ANOVA of different objective functions.

	Source of Variation	Makespan		Total Flow Time		Tardiness	
Main Effects		F	*p*	F	*p*	F	*p*
A: Passion rate		4.23	0.016	19.83	0.000	17.32	0.000
B: number of jobs		30.78	0.000	25.24	0.000	16.32	0.000
C: Tightness		0.07	0.797	0.00	0.996	0.02	0.895

## Data Availability

Not applicable.
